# Unlocking the potential of natural products in drug discovery

**DOI:** 10.1111/1751-7915.13351

**Published:** 2018-12-19

**Authors:** Gerard D. Wright

**Affiliations:** ^1^ M.G. DeGroote Institute for Infectious Disease Research Department of Biochemistry and Biomedical Sciences DeGroote School of Medicine McMaster University 1280 Main Street West Hamilton ON L8S 4K1 Canada

## Abstract

The natural product specialized metabolites produced by microbes and plants are the backbone of our current drugs. Despite their historical importance, few pharmaceutical companies currently emphasize their exploitation in new drug discovery and instead favour synthetic compounds as more tractable alternatives. Ironically, we are in a Golden Age of understanding of natural product biosynthesis, biochemistry and engineering. These advances have the potential to usher in a new era of natural product exploration and development taking full advantage of the unique and favourable properties of natural products compounds in drug discovery.

For millennia, humans have turned to the natural world surrounding them for medicines to treat a range of conditions from infections to gastric disorders to pain management to psychiatric syndromes. These traditional medicines were passed down through generations, often via selected healers in the community, and were the result of anecdote, accumulated experience and custom. Most of these medicines were mixtures of plant products that either were consumed or applied directly, or which were processed, for example through extraction of active ingredients using water (teas) or alcohol (tinctures), or even fermented. Teachings describing the production of many of these medicines formed the foundation of pharmacological knowledge for millennia – elements of traditional Chinese medicine were recorded as early as 1100 BCE (Leung, 2006). Retrospective analysis of some of these methods reveals remarkable efficacy. For example, a recent report evaluating an ancient botanical remedy for eye infections recorded 1,000 years ago in Bald's Leechbook, established antibiotic activity against that pathogen *Staphylococcus aureus* (Harrison *et al*., 2015). The 19th Century saw the first systematic efforts to isolate, purify and chemically analyse the medically active ingredients from plant‐derived traditional medicines. Some of the first such compounds included the anti‐malarial quinine, the analgesic salicylic acid and the opiate morphine.

With the discovery of microbes and the ability to culture them in the lab, plant‐derived medicines were complemented with natural products derived from microorganisms early in the 20th Century. Microbial natural products have proven especially effective as antibiotics and antifungals, e.g., penicillin, amphotericin, etc., but also as anticancer drugs, e.g. doxorubicin, immune‐suppressing agents, e.g. rapamycin, cholesterol‐lowering medicines, e.g. lovastatin, and antiparasitics, e.g., avermectin (Walsh and Tang, 2017). Natural products, their semi‐synthetic derivatives, and synthetic compounds inspired by natural products now form the majority of drugs in use for humans and animals. Antibiotics, in particular, are dominated by the chemical scaffolds derived from microbial natural products.

Why are natural products privileged for clinically useful bioactivity? They are genetically encoded by their producing organisms and as such are the products of evolution through natural selection. This means that they are purposefully made to provide an advantage to the producer. It follows that this advantage is the result of effective engagement of the compound(s) with their cognate biologically relevant receptors or targets. Natural products, therefore, imbue physical chemical characteristics that are necessary for biological activity. Comparison of natural products with chemically synthesized molecules reflects characteristics enriched for active target engagement including increased numbers of *sp* ^*3*^‐hybridized carbons and of chiral centres, fewer aromatic rings, larger macrocyclic aliphatic rings, lower nitrogen content and increased oxygen content, all contributing to more complex three‐dimensional structures. These properties enable natural products to more productively engage biological targets rather than the more planar and less stereochemically complex features that dominate in synthetic compound libraries (Rodrigues *et al*., 2016). The beneficial chemical characteristics of natural products can be further elaborated in the lab though medical chemical strategies to produce compounds with improved drug‐like properties. These include stability to host biochemistry and targeting of specific tissues, and extension of the receptor range beyond those targeted by natural selection.

Despite their proven efficacy in drug discovery and development, the past 30 years have seen a steady decline in the number of new natural product‐derived medicines entering into clinical use. This is the result of many factors. Changes in the process of drug discovery in the pharmaceutical industry in the 1980s and 90s saw a shift to automated assays enabling high throughput screening of thousands to millions of drug candidates. This change required equal access to vast libraries of compounds that could be created with new synthetic strategies such as combinatorial chemistry, but not by traditional natural product extracts of plant or microbiological samples. Another difficulty with natural products is that they require tedious and resource intensive purification to identify the active compounds from complex mixtures, often in tiny amounts. Perhaps the most challenging barrier to natural products over the past few decades is the fact that over 200 000 such compounds have been reported in the scientific literature (Walsh and Tang, 2017). This means that very often, assays for biological activity using traditional natural product extracts identify known rather than novel compounds. Eliminating these known entities, a process termed dereplication can be very resource and time‐consuming. These difficulties conspired to dim the prospects of natural products in drug discovery.

Paradoxically, while the pharmaceutical industry ramped down natural products efforts, the fundamental science of the biosynthesis of these compounds has witnessed an unprecedented series of breakthroughs. First, the biochemical rulebooks that govern the production non‐ribosomal peptides, polyketides, terpenes, alkaloids and others are increasingly becoming clear enabling informed modification and manipulation. Second, the genomic era – where it is straightforward to rapidly and cost‐effectively sequence the whole genome of microbes and other natural product producers – has revealed the myriad genes involved in natural product biosynthesis and shown that, at least in microbes, these genes are assembled contiguously on the genome in clusters that facilitate identification. Consequently, we can identify natural product biosynthetic genes from genome sequences, through algorithms such as antiSMASH (Blin *et al*., 2017), and even predict (though so far with poor accuracy) the structures of produced compounds. What is evident from this growing body of genome sequences is that traditional extraction of natural products followed by purification of compounds has significantly underestimated the diversity of natural products that can be produced. This revelation has the potential to be a game‐changer for natural products in medicine over the next decades (Wright, 2017).

## Mining available genomes

The availability of thousands of genome sequences for natural product producing bacteria is changing the chemical diversity landscape. We now know that in addition to the biosynthetic genes encoding known compounds, each genome reveals many more that have not yet been studied. Each actinomycete bacterial genome has between 20 and 40 clusters while fungal genomes have many more. Plants can contain a dizzying number of biosynthetic genes. As both accurate short read next‐generation genome sequence is increasingly complemented with longer read platforms (PacBio, Nanopore), this biochemical potential is coming much more into focus. Biosynthetic gene clusters are easily identified using a variety of bioinformatic platforms, and prediction of compound structure is improving. Steady improvements in these algorithms will offer an entirely new vista for natural product chemists.

However, this growing understanding biosynthetic capacity is often highly challenging to access in the lab under normal growth conditions. Many of these clusters encoding predicted new chemical scaffolds are inaccessible as a result. There are several ways that the full chemical potential of an organism can be mined. These include the engineering of producing organisms through the expression or deletion of regulatory genes that act directly at the level of cluster expression or even more global regulators that can unlock many pathways (Chen *et al*., 2016). An orthogonal approach is the use of chemical elicitors added to growing organisms that can stimulate compound production, for example by shifting metabolic profiles (Pimentel‐Elardo *et al*., 2015). In yet another strategy, the capture of even very large clusters (> 100 kb) and their cloning into surrogate hosts that may improve production is increasingly commonplace (Xu and Wright, 2018).

These approaches offer several routes to access cryptic or silent gene clusters. A strategic approach would include the careful selection by bioinformatic means of clusters predicted to produce very novel scaffolds. These could be mobilized into a series of surrogate hosts and coupled with genetic efforts to combinatorically explore the impact of regulatory gene expression while in parallel exploring chemical elicitors. Such an approach has the potential to generate libraries of new compounds that can be tested for biological activity using a variety of assays.

## Searching for new natural product producing organisms

In addition to mining organisms that we can readily access and for which we have much experience such as soil microbes, it is apparent that exploring new sources of biosynthetic genes can result in access to new chemical scaffolds. Organisms such as microbial symbionts of sponges, the microbiomes of complex organisms, insect‐associated fungi and bacteria, lichens, and many species of plants have largely been untapped in a systematic fashion as sources of new natural products. Furthermore, the need to isolate and culture individual organisms is no longer a prerequisite to access natural product biosynthetic genes. Instead, metagenomes can be successfully mined for clusters encoding new chemicals in a culture independent fashion (Katz *et al*., 2016). As long read genome sequencing technology improves, such metagenomes may be the preferred sources of new natural product that can be produced using the strategies described above.

## The opportunities for synthetic biology to produce ‘unnatural’ natural products

The ability to both sequence and synthesize DNA on a large scale means that direct engineering of natural products is possible (Wright, 2014). Such synthetic biology tools can be used to fully *de novo* generate previously intractable biosynthetic clusters that do not respond to the expanding repertoire of techniques to coax new compound production in native or orthogonal hosts. Complete refactoring of clusters is now possible, and in so doing, these can be optimized for a variety of hosts from yeast to common bacteria (Smanski *et al*., 2016). Chemical diversity can also be expanded through combinatorialization of biosynthetic genes. Supplemented with optimized fermentation conditions where synthetic chemical precursors can be added, such an approach holds great promise not only to reconstitute pathways but even to generate ‘unnatural’ natural products.

## Conclusions

Natural products have proven to be outstanding sources of medicines but over the past few decades have increasingly been abandoned by the pharmaceutical industry. The strategies outlined above offer several routes to continue to explore these resources to the benefit of all. Advances in genomics, along with increasingly sophisticated gene mining and manipulation, augmented by synthetic biology strategies can be combined to usher in a renaissance in natural product research and development. The possibilities are exciting and the available chemical diversity dizzyingly rich. The path is not easy nor is it the only way forward for new drug discovery. However, natural products do offer unique and specialized chemical space that is uniquely tuned to impact biological process that we should not abandon now that the low hanging fruit has been plucked.
